# Supplementary material for: A Likelihood Approach for Real-Time Calibration of Stochastic Compartmental Epidemic Models
Source: PLoS Comput Biol. 2017 Jan 17;13(1):e1005257. doi: 10.1371/journal.pcbi.1005257 (PMC5240920; doi:10.1371/journal.pcbi.1005257)
Supplement: S1 File — (TAR.GZ) [file pcbi.1005257.s014.tar.gz › HSPH_Online-SI-Revision/EnKF07/n1-severe/n1-severe_EnKF2_table4.pdf]

1	newly 3 weeks spec	24.	29.93	{0., 109.}	150.375	0.887168	newly 3 weeks cumul	81.	141.31	{11., 353.}	158.037	0.470394	newly 5 weeks spec	28.	13.13	{0., 40.}	102.25	1.43691	newly 5 weeks cumul	135.	172.33	{11., 597.}	134.126	0.567539	newly 50 weeks cumul	286.	200.37	{11., 785.}	104.871	0.772588
2	newly 3 weeks spec	23.	10.03	{0., 18.}	82.2174	0.936654	newly 3 weeks cumul	90.	133.87	{10., 185.}	129.544	0.44516	newly 5 weeks spec	19.	2.97	{0., 8.}	88.3684	1.54991	newly 5 weeks cumul	138.	142.18	{12., 197.}	107.435	0.533121	newly 52 weeks cumul	204.	150.88	{13., 303.}	95.4608	0.629352
3	newly 3 weeks spec	38.	29.32	{1., 89.}	104.211	0.823891	newly 3 weeks cumul	102.	200.03	{21., 772.}	169.108	0.478897	newly 5 weeks spec	22.	9.54	{0., 16.}	98.4545	1.20677	newly 5 weeks cumul	152.	225.79	{21., 797.}	140.743	0.551353	newly 51 weeks cumul	261.	237.19	{21., 876.}	108.303	0.677879
4	newly 3 weeks spec	24.	14.35	{0., 45.}	100.625	0.963609	newly 3 weeks cumul	94.	98.62	{9., 231.}	116.596	0.568603	newly 5 weeks spec	9.	4.78	{0., 9.}	103.778	1.18839	newly 5 weeks cumul	116.	110.99	{10., 319.}	112.957	0.607815	newly 54 weeks cumul	163.	120.19	{12., 368.}	103.172	0.697451
5	newly 3 weeks spec	23.	25.18	{0., 86.}	130.	0.894825	newly 3 weeks cumul	56.	135.86	{11., 398.}	202.179	0.474044	newly 5 weeks spec	17.	10.09	{0., 17.}	113.706	1.34365	newly 5 weeks cumul	100.	161.01	{11., 601.}	148.07	0.546697	newly 53 weeks cumul	148.	180.67	{11., 687.}	128.196	0.634376
6	newly 3 weeks spec	43.	12.3	{0., 13.}	105.628	1.93219	newly 3 weeks cumul	120.	81.29	{4., 148.}	110.642	0.868159	newly 5 weeks spec	35.	3.22	{0., 5.}	95.4857	2.21729	newly 5 weeks cumul	192.	90.93	{4., 176.}	101.266	1.01557	newly 50 weeks cumul	340.	101.41	{4., 205.}	96.3147	1.22658
7	newly 3 weeks spec	17.	8.42	{0., 22.}	80.2353	0.867948	newly 3 weeks cumul	65.	87.07	{5., 115.}	118.015	0.486225	newly 5 weeks spec	9.	2.76	{0., 9.}	89.1111	1.31031	newly 5 weeks cumul	90.	93.99	{7., 129.}	101.989	0.539943	newly 50 weeks cumul	125.	106.95	{7., 212.}	99.288	0.640714
8	newly 3 weeks spec	6.	6.49	{0., 10.}	110.5	0.700531	newly 3 weeks cumul	53.	92.53	{8., 90.}	154.925	0.432613	newly 5 weeks spec	0.1	2.46	{0., 4.}	2488.	0.516109	newly 5 weeks cumul	55.	98.79	{8., 116.}	158.018	0.433756	newly 54 weeks cumul	55.	104.1	{9., 131.}	166.	0.429166
9	newly 3 weeks spec	21.	13.03	{0., 24.}	105.857	1.08505	newly 3 weeks cumul	83.	67.43	{6., 147.}	75.6747	0.483761	newly 5 weeks spec	22.	4.21	{0., 8.}	95.5909	1.75335	newly 5 weeks cumul	119.	79.63	{6., 174.}	77.8571	0.570409	newly 50 weeks cumul	236.	85.13	{6., 179.}	78.7924	0.79167
10	newly 3 weeks spec	16.	24.77	{1., 63.}	160.563	0.690776	newly 3 weeks cumul	74.	103.7	{12., 169.}	131.649	0.48607	newly 5 weeks spec	16.	14.15	{0., 44.}	127.938	1.12884	newly 5 weeks cumul	103.	135.9	{15., 345.}	132.757	0.543826	newly 50 weeks cumul	137.	174.45	{17., 798.}	135.73	0.602297
11	newly 3 weeks spec	26.	30.95	{0., 153.}	170.038	1.41549	newly 3 weeks cumul	70.	110.02	{6., 484.}	164.229	0.648651	newly 5 weeks spec	28.	7.41	{0., 12.}	100.821	1.86314	newly 5 weeks cumul	115.	132.06	{6., 678.}	141.461	0.778621	newly 47 weeks cumul	274.	141.66	{6., 744.}	102.321	1.03307
12	newly 3 weeks spec	14.	7.93	{0., 11.}	99.5	1.12479	newly 3 weeks cumul	54.	82.05	{5., 137.}	131.315	0.437982	newly 5 weeks spec	7.	2.45	{0., 5.}	99.8571	1.41321	newly 5 weeks cumul	71.	88.73	{6., 151.}	119.028	0.49747	newly 55 weeks cumul	74.	96.21	{6., 187.}	122.284	0.503558
13	newly 3 weeks spec	28.	10.56	{0., 13.}	112.071	1.78075	newly 3 weeks cumul	97.	58.07	{4., 71.}	102.814	0.780074	newly 5 weeks spec	15.	1.75	{0., 3.}	97.1333	1.88482	newly 5 weeks cumul	134.	64.13	{5., 78.}	100.291	0.876684	newly 50 weeks cumul	214.	66.5	{6., 78.}	94.7664	1.03372
14	newly 3 weeks spec	29.	14.13	{0., 36.}	97.4828	1.16616	newly 3 weeks cumul	89.	97.38	{6., 263.}	116.427	0.600474	newly 5 weeks spec	15.	4.22	{0., 8.}	96.9333	1.50953	newly 5 weeks cumul	126.	109.7	{7., 286.}	107.381	0.677723	newly 55 weeks cumul	170.	115.56	{7., 316.}	99.3647	0.7575
15	newly 3 weeks spec	13.	21.64	{2., 62.}	137.077	0.463552	newly 3 weeks cumul	54.	127.91	{14., 286.}	177.278	0.342748	newly 5 weeks spec	3.	7.12	{0., 18.}	222.667	0.747783	newly 5 weeks cumul	64.	147.29	{15., 390.}	172.859	0.347598	newly 49 weeks cumul	74.	156.82	{15., 420.}	160.081	0.35547
16	newly 3 weeks spec	18.	16.29	{0., 40.}	121.056	0.999431	newly 3 weeks cumul	97.	136.38	{5., 416.}	150.309	0.663331	newly 5 weeks spec	13.	4.71	{0., 8.}	98.0769	1.40384	newly 5 weeks cumul	125.	149.83	{5., 463.}	136.568	0.711398	newly 55 weeks cumul	201.	156.35	{5., 486.}	110.512	0.827079
17	newly 3 weeks spec	13.	13.28	{0., 25.}	125.692	0.850399	newly 3 weeks cumul	54.	71.65	{7., 151.}	131.093	0.532791	newly 5 weeks spec	4.	6.73	{0., 9.}	188.25	0.873263	newly 5 weeks cumul	73.	87.82	{8., 171.}	126.055	0.572821	newly 55 weeks cumul	87.	107.76	{9., 171.}	128.828	0.585193
18	newly 3 weeks spec	19.	8.96	{0., 13.}	97.6842	1.18121	newly 3 weeks cumul	61.	70.1	{8., 124.}	104.557	0.447609	newly 5 weeks spec	28.	2.37	{0., 6.}	93.6071	1.79341	newly 5 weeks cumul	119.	77.7	{9., 126.}	91.1092	0.628736	newly 55 weeks cumul	388.	82.68	{12., 126.}	84.3299	1.01857
19	newly 3 weeks spec	15.	5.79	{0., 10.}	100.733	1.42027	newly 3 weeks cumul	71.	107.22	{2., 400.}	164.225	0.704211	newly 5 weeks spec	12.	1.06	{0., 1.}	96.1667	1.81041	newly 5 weeks cumul	93.	110.39	{2., 419.}	143.688	0.778644	newly 48 weeks cumul	186.	112.45	{2., 419.}	108.758	0.996767
20	newly 3 weeks spec	7.	8.51	{0., 31.}	157.286	1.10184	newly 3 weeks cumul	54.	57.63	{4., 112.}	117.352	0.609572	newly 5 weeks spec	3.	3.24	{0., 13.}	156.667	1.16408	newly 5 weeks cumul	64.	65.46	{4., 153.}	117.844	0.65113	newly 48 weeks cumul	97.	74.84	{4., 253.}	107.443	0.775861
21	newly 3 weeks spec	33.	15.6	{0., 14.}	105.515	1.18732	newly 3 weeks cumul	107.	65.24	{6., 67.}	101.57	0.747116	newly 5 weeks spec	12.	8.34	{0., 14.}	124.667	1.21774	newly 5 weeks cumul	135.	84.09	{7., 74.}	105.578	0.77512	newly 55 weeks cumul	223.	112.64	{8., 249.}	103.175	0.898399
22	newly 3 weeks spec	12.	16.34	{0., 41.}	156.833	0.975603	newly 3 weeks cumul	57.	88.86	{5., 245.}	147.86	0.542287	newly 5 weeks spec	7.	9.03	{0., 23.}	175.	1.34908	newly 5 weeks cumul	75.	109.72	{5., 364.}	146.213	0.607269	newly 49 weeks cumul	81.	126.23	{5., 417.}	157.346	0.639155
23	newly 3 weeks spec	21.	17.23	{0., 56.}	121.952	1.18558	newly 3 weeks cumul	88.	128.58	{2., 423.}	149.364	0.686381	newly 5 weeks spec	16.	5.97	{0., 18.}	102.188	1.57049	newly 5 weeks cumul	122.	144.51	{2., 459.}	132.861	0.763355	newly 51 weeks cumul	188.	163.73	{2., 502.}	114.612	0.883736
24	newly 3 weeks spec	15.	7.19	{0., 8.}	105.933	1.31082	newly 3 weeks cumul	68.	105.49	{9., 169.}	141.779	0.46292	newly 5 weeks spec	6.	0.66	{0., 1.}	93.	1.50623	newly 5 weeks cumul	79.	108.25	{9., 173.}	131.81	0.497594	newly 48 weeks cumul	103.	108.58	{9., 173.}	117.146	0.575888
25	newly 3 weeks spec	9.	6.06	{0., 13.}	95.7778	0.845356	newly 3 weeks cumul	59.	48.98	{8., 92.}	82.3051	0.467031	newly 5 weeks spec	11.	2.44	{0., 3.}	103.091	1.67566	newly 5 weeks cumul	83.	54.83	{8., 98.}	81.6988	0.554971	newly 46 weeks cumul	100.	66.48	{8., 103.}	89.98	0.617254
26	newly 3 weeks spec	16.	12.68	{0., 18.}	121.875	1.20006	newly 3 weeks cumul	56.	118.25	{9., 323.}	197.446	0.500774	newly 5 weeks spec	15.	6.67	{0., 7.}	116.333	1.62111	newly 5 weeks cumul	84.	133.72	{9., 536.}	161.952	0.575184	newly 53 weeks cumul	169.	154.11	{9., 723.}	122.858	0.76
27	newly 3 weeks spec	8.	19.74	{2., 49.}	199.5	0.430486	newly 3 weeks cumul	28.	113.58	{14., 299.}	325.929	0.415984	newly 5 weeks spec	0.1	6.92	{0., 16.}	6872.	1.23267	newly 5 weeks cumul	28.	131.95	{17., 335.}	386.75	0.457586	newly 52 weeks cumul	28.	144.65	{19., 351.}	429.679	0.49851
28	newly 3 weeks spec	36.	25.22	{1., 75.}	100.444	0.873101	newly 3 weeks cumul	124.	162.86	{16., 490.}	123.419	0.550105	newly 5 weeks spec	31.	6.64	{0., 15.}	90.8387	1.52194	newly 5 weeks cumul	183.	182.95	{16., 546.}	107.809	0.624532	newly 55 weeks cumul	256.	194.93	{16., 557.}	99.4414	0.717796
29	newly 3 weeks spec	15.	10.14	{0., 17.}	87.6	0.733087	newly 3 weeks cumul	52.	50.29	{4., 77.}	89.0577	0.513975	newly 5 weeks spec	10.	4.87	{0., 8.}	95.3	1.13143	newly 5 weeks cumul	71.	62.44	{4., 98.}	90.0282	0.560083	newly 48 weeks cumul	150.	78.69	{5., 130.}	87.86	0.777304
30	newly 3 weeks spec	15.	14.3	{0., 33.}	116.533	0.814711	newly 3 weeks cumul	70.	103.98	{15., 173.}	105.286	0.347073	newly 5 weeks spec	2.	4.87	{0., 7.}	270.5	0.978163	newly 5 weeks cumul	78.	116.65	{15., 202.}	110.782	0.367895	newly 49 weeks cumul	112.	123.53	{15., 272.}	100.223	0.462523
31	newly 3 weeks spec	19.	15.21	{0., 21.}	120.474	1.17296	newly 3 weeks cumul	55.	111.32	{16., 148.}	155.236	0.34628	newly 5 weeks spec	24.	8.71	{0., 24.}	101.042	1.58802	newly 5 weeks cumul	98.	132.28	{18., 224.}	122.265	0.447832	newly 50 weeks cumul	218.	156.91	{20., 285.}	99.9495	0.670851
32	newly 3 weeks spec	37.	4.01	{0., 8.}	93.8649	1.94267	newly 3 weeks cumul	98.	95.58	{4., 307.}	130.49	0.739673	newly 5 weeks spec	27.	0.69	{0., 1.}	97.6667	2.27973	newly 5 weeks cumul	151.	97.68	{4., 307.}	111.894	0.871734	newly 52 weeks cumul	280.	98.72	{4., 307.}	95.6929	1.07207
33	newly 3 weeks spec	31.	15.44	{0., 44.}	103.742	1.19795	newly 3 weeks cumul	119.	75.75	{8., 179.}	100.143	0.724861	newly 5 weeks spec	13.	5.72	{0., 13.}	102.	1.39254	newly 5 weeks cumul	150.	90.53	{10., 230.}	99.58	0.760725	newly 55 weeks cumul	184.	105.61	{10., 355.}	97.0489	0.79254
34	newly 3 weeks spec	15.	14.4	{0., 24.}	152.	1.47259	newly 3 weeks cumul	64.	113.4	{7., 409.}	163.688	0.544227	newly 5 weeks spec	10.	7.46	{0., 8.}	147.2	1.68589	newly 5 weeks cumul	87.	131.21	{7., 461.}	152.31	0.618154	newly 47 weeks cumul	179.	150.55	{7., 467.}	113.246	0.800657
35	newly 3 weeks spec	21.	18.82	{0., 19.}	133.619	1.0167	newly 3 weeks cumul	87.	92.11	{7., 284.}	124.471	0.646075	newly 5 weeks spec	3.	8.54	{0., 12.}	292.667	0.852626	newly 5 weeks cumul	98.	112.62	{8., 386.}	131.878	0.644998	newly 55 weeks cumul	98.	129.16	{8., 390.}	143.082	0.6267
36	newly 3 weeks spec	1.	11.5	{0., 15.}	1086.	0.7542	newly 3 weeks cumul	11.	105.85	{7., 108.}	877.	0.607313	newly 5 weeks spec	0.1	2.4	{0., 4.}	2436.	0.486434	newly 5 weeks cumul	11.	113.42	{7., 127.}	945.273	0.630378	newly 56 weeks cumul	11.	115.8	{7., 141.}	966.909	0.64092
37	newly 3 weeks spec	19.	16.16	{0., 40.}	131.789	1.1199	newly 3 weeks cumul	60.	126.45	{6., 386.}	197.317	0.522362	newly 5 weeks spec	11.	2.82	{0., 4.}	100.909	1.62968	newly 5 weeks cumul	82.	135.78	{6., 425.}	170.585	0.606465	newly 46 weeks cumul	171.	141.42	{6., 431.}	122.55	0.842204
38	newly 3 weeks spec	5.	10.75	{0., 13.}	198.2	0.694816	newly 3 weeks cumul	41.	96.62	{3., 127.}	198.732	0.457643	newly 5 weeks spec	3.	2.69	{0., 5.}	133.	1.02462	newly 5 weeks cumul	46.	104.66	{3., 134.}	192.13	0.469398	newly 54 weeks cumul	51.	109.62	{3., 134.}	184.314	0.491859
39	newly 3 weeks spec	20.	2.27	{0., 6.}	93.15	1.73899	newly 3 weeks cumul	64.	46.32	{4., 63.}	95.625	0.625718	newly 5 weeks spec	14.	0.31	{0., 0.}	97.7857	2.0189	newly 5 weeks cumul	91.	47.63	{4., 64.}	91.8132	0.742975	newly 50 weeks cumul	151.	48.04	{4., 64.}	89.5894	0.93033
40	newly 3 weeks spec	21.	5.77	{0., 14.}	73.8571	0.923507	newly 3 weeks cumul	63.	39.33	{8., 70.}	52.873	0.41644	newly 5 weeks spec	9.	2.58	{0., 7.}	80.	1.17513	newly 5 weeks cumul	89.	45.84	{9., 84.}	57.1011	0.488344	newly 56 weeks cumul	118.	53.35	{10., 102.}	62.5169	0.566598
41	newly 3 weeks spec	14.	29.19	{0., 121.}	218.929	0.876088	newly 3 weeks cumul	52.	143.84	{9., 479.}	249.231	0.543824	newly 5 weeks spec	6.	8.75	{0., 24.}	184.833	1.16758	newly 5 weeks cumul	63.	169.44	{9., 721.}	244.825	0.558022	newly 53 weeks cumul	66.	182.22	{9., 842.}	250.97	0.563174
42	newly 3 weeks spec	25.	22.36	{0., 42.}	130.48	1.21603	newly 3 weeks cumul	82.	128.39	{11., 364.}	139.329	0.511225	newly 5 weeks spec	15.	5.4	{0., 10.}	103.333	1.58306	newly 5 weeks cumul	118.	144.94	{13., 401.}	124.186	0.58861	newly 53 weeks cumul	257.	150.3	{13., 401.}	96.3813	0.81765
43	newly 3 weeks spec	9.	7.5	{0., 9.}	145.778	1.44607	newly 3 weeks cumul	28.	68.69	{3., 54.}	197.464	0.402059	newly 5 weeks spec	9.	2.15	{0., 0.}	112.111	1.81406	newly 5 weeks cumul	44.	75.35	{3., 58.}	158.477	0.505195	newly 46 weeks cumul	120.	76.41	{3., 58.}	110.442	0.85569
44	newly 3 weeks spec	16.	8.11	{0., 14.}	93.8125	0.991279	newly 3 weeks cumul	64.	66.3	{8., 154.}	99.6563	0.482355	newly 5 weeks spec	5.	3.52	{0., 6.}	112.	1.02621	newly 5 weeks cumul	85.	75.15	{9., 154.}	95.7529	0.538951	newly 54 weeks cumul	109.	81.59	{9., 154.}	92.8349	0.602243
45	newly 3 weeks spec	17.	17.27	{1., 30.}	86.5294	0.518475	newly 3 weeks cumul	55.	98.1	{10., 114.}	125.855	0.371076	newly 5 weeks spec	8.	6.74	{0., 16.}	93.	0.860172	newly 5 weeks cumul	80.	114.71	{11., 143.}	102.188	0.390482	newly 51 weeks cumul	157.	134.47	{12., 214.}	83.586	0.528028
46	newly 3 weeks spec	36.	5.07	{0., 12.}	93.1944	1.79724	newly 3 weeks cumul	87.	99.85	{7., 126.}	119.138	0.569433	newly 5 weeks spec	13.	1.78	{0., 3.}	97.2308	1.86017	newly 5 weeks cumul	135.	103.81	{7., 152.}	102.526	0.692642	newly 50 weeks cumul	318.	111.7	{7., 160.}	92.3774	0.993331
47	newly 3 weeks spec	0.1	20.55	{0., 20.}	20504.	1.33125	newly 3 weeks cumul	20.	89.44	{4., 152.}	378.5	0.479726	newly 5 weeks spec	0.1	6.49	{0., 11.}	6498.	0.75609	newly 5 weeks cumul	20.	107.67	{4., 155.}	467.05	0.51037	newly 56 weeks cumul	20.	114.37	{4., 163.}	498.55	0.522005
48	newly 3 weeks spec	16.	24.56	{0., 57.}	151.25	0.696022	newly 3 weeks cumul	39.	109.84	{11., 314.}	224.	0.425995	newly 5 weeks spec	12.	9.64	{0., 30.}	116.	1.13385	newly 5 weeks cumul	57.	135.26	{12., 333.}	194.842	0.459058	newly 50 weeks cumul	102.	158.81	{12., 463.}	143.912	0.5609
49	newly 3 weeks spec	13.	10.9	{0., 11.}	118.769	0.920032	newly 3 weeks cumul	64.	91.51	{13., 237.}	141.078	0.47856	newly 5 weeks spec	9.	3.11	{0., 4.}	99.4444	1.28208	newly 5 weeks cumul	90.	100.44	{15., 350.}	124.8	0.551265	newly 54 weeks cumul	127.	106.03	{16., 362.}	111.567	0.646904
50	newly 3 weeks spec	21.	6.19	{0., 10.}	97.5714	1.38929	newly 3 weeks cumul	56.	68.09	{5., 136.}	110.411	0.492816	newly 5 weeks spec	17.	1.54	{0., 2.}	96.4706	1.96607	newly 5 weeks cumul	91.	72.33	{5., 136.}	97.044	0.623938	newly 52 weeks cumul	172.	73.87	{5., 136.}	88.5058	0.827805
